# Prevalence and severity of apical root resorption during orthodontic treatment with clear aligners and fixed appliances: a cone beam computed tomography study

**DOI:** 10.1186/s40510-019-0301-1

**Published:** 2020-01-06

**Authors:** Yuan Li, Shiyong Deng, Li Mei, Zhengzheng Li, Xinyun Zhang, Chao Yang, Yu Li

**Affiliations:** 1 State Key Laboratory of Oral Diseases & National Clinical Research Center for Oral Diseases, West China Hospital of Stomatology, Sichuan University, Chengdu, China; 2 Discipline of Orthodontics, Department of Oral Sciences, Sir John Walsh Research Institute, Faculty of Dentistry, University of Otago, Dunedin, New Zealand; 3 Department of Epidemiology and Health statistics, School of Public Health, Southwest Medical University, Luzhou, China

**Keywords:** Root resorption, Invisalign, Clear aligners, Braces, CBCT

## Abstract

**Background:**

Fixed appliances have been the mainstream for orthodontic treatment, while clear aligners, such as Invisalign system, have become increasingly popular. The prevalence of apical root resorption (ARR) in patients with clear aligners is still controversial. The aim of this study was to investigate and compare the prevalence and severity of ARR in patients treated with clear aligners and fixed appliances using cone beam computed tomography (CBCT).

**Materials and methods:**

A total of 373 roots from 70 subjects, with similar baseline characteristics and the ABO discrepancy index scores (i.e., treatment difficulty), were included into two groups: the clear aligners group (Invisalign, Align Technology, California, USA) and fixed appliances group (Victory Series; 3 M Unitek, California, USA). Root length of each anterior tooth was measured on the CBCT images by two blinded investigators. The ARR on each tooth was calculated as the difference of root length before and after orthodontic treatment. Chi-square test and paired *t* test was used to compare the ARR between the two groups as well as before and after orthodontic treatments.

**Results:**

Prevalence of ARR in the clear aligners group (56.30%) was significantly lower than that in the fixed appliances group (82.11%) (*P* < 0.001). The severity of ARR in the clear aligners group (0.13 ± 0.47 mm) was significantly less than that in the fixed appliances group (1.12 ± 1.34 mm) (*P* < 0.001). The most severe ARR was found on the maxillary canine (1.53 ± 1.92 mm) and lateral incisor (1.31 ± 1.33 mm) in the fixed appliances group; the least ARR was found on the mandibular canine (− 0.06 ± 0.47 mm) and lateral incisor (0.04 ± 0.48 mm) in the clear aligners group (*P* < 0.001).

**Conclusions:**

The prevalence and severity of ARR measured on CBCT in patients with clear aligners were less than those in patients with fixed appliances.

## Background

Apical root resorption (ARR), a permanent loss of hard tissue on the root apex of a tooth, is one of the most undesirable side effects during orthodontic treatment. The prevalence of ARR varies from 20 to 100% among orthodontic patients [1]. Severe ARR is rare with an incidence between 1 and 5% but the resorption can be more than 5 mm or one-fourth of root length [2]. ARR can cause an imbalanced ratio of crown and root in the affected teeth, and even teeth loss, affecting patients’ quality of life and orthodontic treatment result.

Fixed appliances have been the mainstream for orthodontic treatment. Clear aligners, such as Invisalign system, have become increasingly popular due to its advantages, such as esthetics and comfort, in comparison with fixed appliances [3, 4]. It has been found that the type of fixed appliances used for orthodontic treatment was associated with the incidence of ARR [5, 6]. The prevalence of ARR in patients with clear aligners is still controversial [7]. For example, a study on clear aligners assessed the upper and lower anterior teeth and first molars using panoramic radiographs and found that 46% of the teeth showed measurable root reduction during the treatment with clear aligners [8]. This prevalence of ARR seems equal to or less than that in fixed appliances [9, 10]. The potential problem is that clear aligners are usually used in relatively simpler cases where root resorption might be expected to be less. Therefore, it would be good that patients treated with either technique must be equal in difficulty, amount of tooth movement required, and outcome quality for treatment and comparison.

In addition, the tools used for assessing ARR in those studies could also influence the accuracy of ARR measurement. For example, a study has compared clear aligners with fixed appliances using panoramic radiography and found a similar ARR predisposition on maxillary central and lateral incisors [11]. However, the study using cone beam computed tomography (CBCT) to measure ARR found that patients with clear aligners suffered from significantly less incisor root resorption than that with fixed appliances [12]. It has been found that panoramic radiography may overestimate the prevalence of ARR by 20% compared with periapical radiography [13], and underestimate compared with microtomography [14]. Since ARR is a three-dimensional topographical change, two-dimensional radiography, such as panoramic and periapical radiographs, have limitations in the accuracy of ARR measurement. In contrast, the three-dimensional radiography, cone beam computed tomography (CBCT), has demonstrated a relatively higher accuracy in diagnosis and measurement of ARR [15, 16].

The aim of the study was to investigate and compare the prevalence and severity of ARR in patients treated with clear aligners and fixed appliances using CBCT.

## Materials and methods

The study was designed as a retrospective cohort study. A total of 373 roots from 70 subjects (mean age 23.61 ± 7.03 years, 21 males and 49 females) were included from the Department of Orthodontics (Table 1). The sample size determination was based on previous estimates of ARR variability in patients wearing fixed appliances [9], with α set at 5%, β at 20%, effect size of 0.8, a total of 52 patients (26 per group) were needed. To allow for possible dropout during the study, we included 70 patients. Ethical approval was obtained from the Ethics Committee of West China Hospital of Stomatology, Sichuan University. Written informed consent was obtained from each participant.

**Table 1:** Baseline characteristics of participants in the two groups

	Clear aligners (*N* = 35)	Fixed appliances (*N* = 35)
Age (mean ± SD)	24.71 ± 7.48	22.51 ± 6.47
Sex (*N*, %)		
Male	13 (37%)	8 (23%)
Female	22 (63%)	27 (77%)
Treatment time (mean ± SD)	21.54 ± 5.55	23.31 ± 6.25
Extraction (*N*, %)	19 (54%)	14 (40%)

*SD* standard deviation

Inclusion criteria were (1) subjects that have full permanent dentition with no missing teeth, (2) no history of major dental treatment, (3) received orthodontic treatment with traditional appliances or clear aligners, and (4) the CBCT images were taken as part of their orthodontic diagnosis and treatment plan and were of good quality. Exclusion criteria were (1) craniofacial defects, syndromes or skeletal deformity (e.g., cleft lip and palate); (2) history of trauma; (3) history or orthodontic or endodontic treatment on the anterior teeth; (4) significant dental pathology affecting anterior teeth, such as root absorption, periodontitis, periodontal diseases, and caries; and (5) supernumerary teeth, impacted teeth (except the third molars), and temporomandibular joint disorders.

**Fig. 1:**
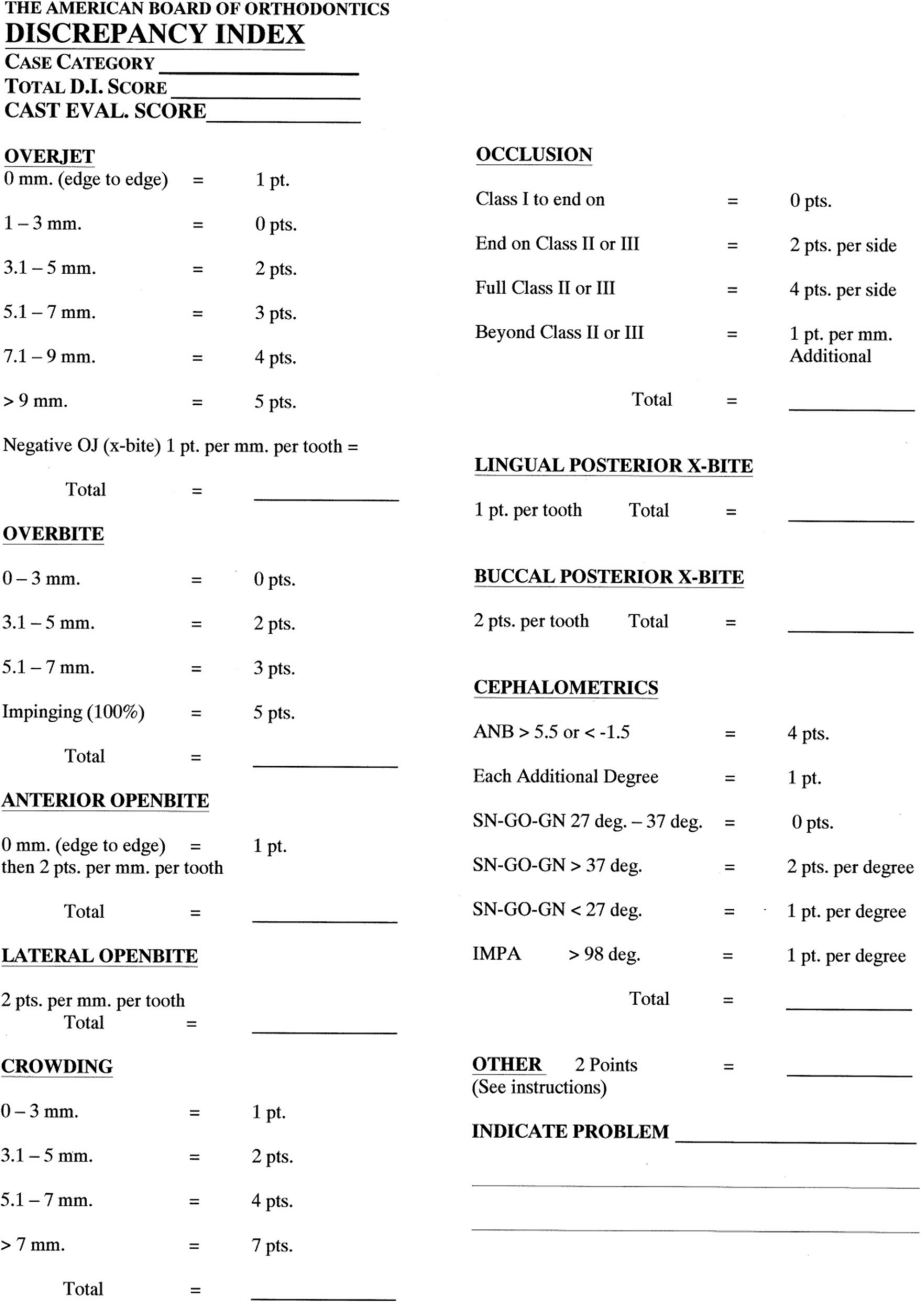
The ABO discrepancy index (DI)

The clear aligners group (*n* = 35) received treatment with clear aligners (Invisalign, Align Technology, California, USA). The fixed appliances group (*n* = 35) received treatment with the conventional fixed orthodontic appliances (Victory Series; 3 M Unitek, California, USA). To maximize the comparability of the two groups, the following variables were taken into account during the screening of 578 patients for eligibility: the severity and type of malocclusion, biomechanics, and amount of tooth movement (e.g., extraction and non-extraction), and treatment outcome quality. The American Board of Orthodontics (ABO) discrepancy index (DI) was used to assess the case difficulty in the two groups (Fig. 1) [17]. Baseline characteristics of the participants in the two groups were all similar (*P* > 0.192 for all) (Table 1). The ABO discrepancy index took into account the overall DI scores of clear aligners group (18.80 ± 7.74) and fixed appliances group (17.14 ± 10.14), and indicating that the baseline difficulty of the two groups was also similar (*P* = 0.445, Table 2).

**Table 2:** Baseline discrepancy index (DI) of the two groups

	Clear aligners	Fixed appliances	*P* value
Overjet	3.02 ± 3.33	2.29 ± 1.07	0.216
Overbite	1.02 ± 1.18	0.46 ± 0.89	0.025
Anterior open bite	0.86 ± 2.35	0.29 ± 1.18	0.205
Lateral open bite	0.06 ± 0.34	1.60 ± 2.86	0.003
Crowding	2.43 ± 2.38	1.31 ± 1.84	0.032
Occlusion	2.40 ± 2.37	1.54 ± 2.06	0.111
Posterior crossbite	0.69 ± 1.64	0.17 ± 0.51	0.084
ANB angle	2.49 ± 2.78	2.29 ± 2.99	0.773
SN-MP angle	1.00 ± 2.28	3.69 ± 5.28	0.008
IMPA angle	4.14 ± 5.85	3.03 ± 3.98	0.355
Total score	18.80 ± 7.74	17.14 ± 10.14	0.445

CBCT images were obtained from all participants before and after orthodontic treatments. All CBCT images were taken using the same CBCT machine (3D Accuitomo, Morita Group, JPN), and the settings used were in accordance with the manufacturers’ recommendations (10 × 10 cm FOV, 85 kV, 4 mA and 360° rotation). During image acquisition, the participants were seated statically with the Frankfort plane parallel to the ground.

**Fig. 2:**
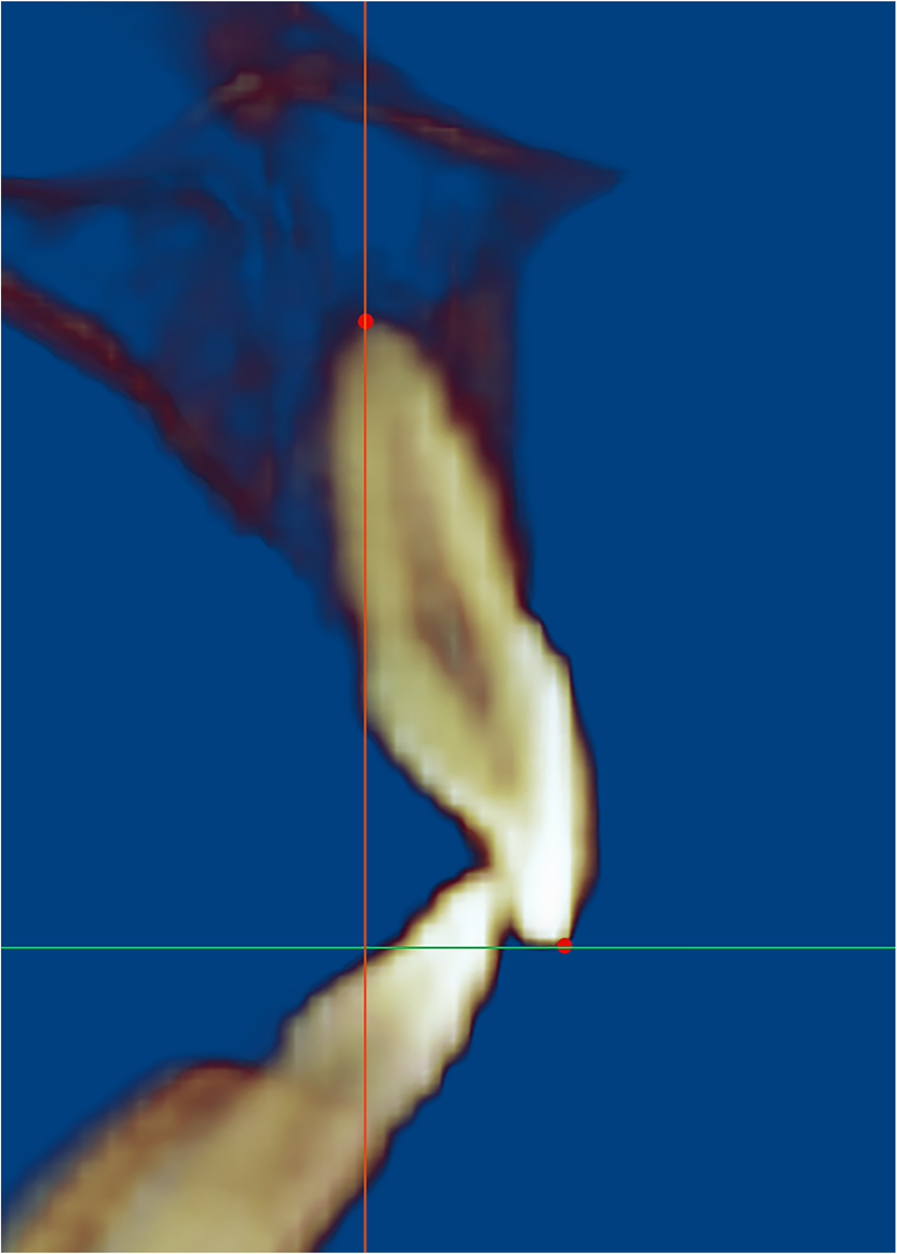
Measurement of apical root resorption (ARR) on CBCT

To assess the apical root resorption (ARR), two blinded dental investigators (Y.L. and S.D.) measured the root length from the mid-point of the incisal edge/cusp to the apex using the Dolphin 3D 11.9 program (Dolphin Imaging & Management Solutions, Chatsworth, CA) (Fig. 2). ARR on each tooth was calculated as the difference (millimeter) of tooth length before and after orthodontic treatment. All maxillary and mandibular anterior teeth were included in the measurements and analysis.

Both intra-operator and inter-operator reliabilities were tested by using intra-class correlation coefficients. Ten CBCT images were randomly selected and measured by the two independent dental investigators; after 4 weeks, each investigator repeated the measurements. The inter-rater reliability was excellent (correlation coefficient was 0.98). The intra-rater reliability was excellent (correlation coefficients for the two investigators were 0.98 and 0.97). The two investigators as well as the statistician were all blinded to the study design.

### Statistical analysis

Data were analyzed using SPSS Statistics 21(Statistical Package for the Social Sciences, SPSS Inc., Chicago, IL, USA). Student’s *t* test was used to compare the baseline characteristics of the two groups. Chi-square test and paired *t* test was used to compare the prevalence and severity of ARR between the two groups as well as before and after orthodontic treatment. *P* values of less than 0.05 were considered statistically significant.

## Results

The prevalence of ARR in the clear aligners group (56.30%) was significantly lower than that in the fixed appliances group (82.11%) (*P* < 0.001, Table 3). The highest prevalence of ARR was found on the lateral incisors in patients with fixed appliances (maxillary 88.52%, mandibular 88.33%), and the lowest was found on the canines in patients with clear aligners (maxillary 45.00%, mandibular 35.38%).

**Table 3:** Prevalence of apical root resorption in the two groups

	Clear aligners	Fixed appliances	*P* value
Maxillary central incisor	69.35%	84.62%	0.041
Maxillary lateral incisor	69.35%	88.52%	0.009
Maxillary canine	45.00%	80.00%	0.001
Mandibular central incisor	60.00%	78.69%	0.023
Mandibular lateral incisor	53.85%	88.33%	< 0.001
Mandibular canine	35.38%	72.58%	< 0.001
Average	56.30%	82.11%	< 0.001

The severity of ARR in clear aligners group (0.13 ± 0.47 mm on average) was significantly less than that in the fixed appliances group (1.12 ± 1.34 mm on average), as well as for each individual tooth included in the study (all *P* < 0.001) (Table 4). In fixed appliances group, there was a statistically significant decrease in the root length of all teeth measured before and after the treatment (*P* < 0.001 for all); while in the clear aligners group, the root length change was statistically significant only on the maxillary incisors (*P* < 0.001) and mandibular central incisor (*P* = 0.001). The most severe ARR was found on the maxillary canine (1.53 ± 1.92 mm) and lateral incisor (1.31 ± 1.33 mm) in the fixed appliances group; the least ARR was found on the mandibular canine (− 0.06 ± 0.47 mm) and lateral incisor (0.04 ± 0.48 mm) in the clear aligners group (*P* < 0.001).

**Table 4:** Severity of apical root resorption of individual tooth in the two groups

Measurements (mean ± SD, mm)	Root length			Apical root resorption	*P* value
	Before treatment	After treatment			
Clear aligners					
Maxillary central incisor	21.55 ± 1.86	21.29 ± 2.01		0.26 ± 0.42	< 0.001
Maxillary lateral incisor	20.60 ± 1.94	20.37 ± 2.02		0.23 ± 0.37	< 0.001
Maxillary canine	23.89 ± 2.63	23.75 ± 2.72		0.14 ± 0.53	0.075
Mandibular central incisor	18.56 ± 1.39	18.36 ± 1.29		0.20 ± 0.45	0.001
Mandibular lateral incisor	19.83 ± 1.40	19.79 ± 1.31		0.04 ± 0.48	0.463
Mandibular canine	22.61 ± 2.37	22.67 ± 2.35		−0.06 ± 0.47	0.289
Average	21.09 ± 2.61	20.96 ± 2.66		0.13 ± 0.47	0.500
Fixed appliances					
Maxillary central incisor	21.25 ± 1.85	20.02 ± 1.94		1.23 ± 1.31	< 0.001
Maxillary lateral incisor	20.23 ± 1.53	18.92 ± 1.97		1.31 ± 1.33	< 0.001
Maxillary canine	23.39 ± 2.19	21.86 ± 2.54		1.53 ± 1.92	< 0.001
Mandibular central incisor	18.26 ± 1.06		17.61 ± 1.26	0.65 ± 0.89	< 0.001
Mandibular lateral incisor	19.52 ± 1.21		18.50 ± 1.27	1.02 ± 0.98	< 0.001
Mandibular canine	21.98 ± 1.71		20.96 ± 2.01	1.02 ± 1.33	< 0.001
Average	20.74 ± 2.31		19.62 ± 2.36	1.12 ± 1.34	< 0.001

To evaluate the clinical significance, the severity of root resorption was categorized based on Sharpe’s method [38]:

0° = No ARR, ARR = 0 mm;

1° = Slight blunting of the root apex, ARR = 1–2 mm;

2° = Moderate blunting of the root apex up to one fourth of the root length, ARR = 2 mm–1/4 root length;

3° = Excessive blunting of the root apex beyond one fourth of the root length, ARR > 1/4 root length.

In the fixed appliances group, 18.16% of the teeth appeared to have 0° ARR, 61.79% appeared to have 1° ARR, 19.24% appeared to be 2° and 0.81% was 3°; while in the clear aligners group, 43.70% displayed 0° ARR and 56.30% was 1°, indicating that the ARR in the fixed appliances group was generally greater than that in the clear aligners group (Table 5).

**Table 5:** Classification of overall severity of apical root resorption (ARR) in the two groups

Severity of ARR	Clear aligners (*N*, %)		Fixed appliances (*N*, %)	
0°	163	43.70%	67	18.16%
1°	210	56.30%	228	61.79%
2°	0	0	71	19.24%
3°	0	0	3	0.81%

## Discussion

Apical root resorption (ARR) during orthodontic treatment is prevalent and negatively affects patients’ quality of life and orthodontic treatment result. Clear aligners have become increasingly popular for orthodontic treatment; ARR during the clear aligners treatment, however, is still poorly understood. This study investigated and compared the ARR in patients treated with clear aligners and traditional fixed appliances using CBCT, and found that both prevalence and severity of ARR in the clear aligners group (56.30% and 0.13 ± 0.47 mm) were statistically and clinically significantly less than those in the fixed appliances group (82.11% and 1.12 ± 1.34 mm).

In comparison with fixed appliances, the clear aligners are usually used in relatively simpler cases where root resorption might be expected to be less. Therefore, in the current study, the baseline characteristics assessment and the ABO discrepancy index (DI) were carried out to make sure that patients were treated with either technique was similar in difficulty, amount of tooth movement required, and outcome quality for treatment and comparison [17].

Apical root resorption occurs mainly in the anterior teeth, and varies in different tooth positions [18]. The current study design of measurement on the anterior teeth is based on the previous studies [5, 12, 18] for the practical convenience and good accuracy of ARR measurement. The maxillary incisors have been found to be the most susceptible to ARR, followed by the mandibular incisors, while canines did not develop significant ARR [9, 19], which are in agreement with the current CBCT study. Interestingly, maxillary canines in the study showed the most ARR (1.53 mm) in fixed appliances group but no significance in the clear aligners group. Generally, ARR during fixed orthodontic treatment is less than 2.5 mm [2, 20]. It has been found that the mean root length loss of maxillary incisors treated with fixed appliances was 2.26 mm on periapical radiographs [5]. Another study used CBCT reported that the maxillary incisor length shortened 0.59 mm in patients treated with fixed appliances [6]. In the current study, the average ARR was found to be 1.12 mm in patients with fixed appliances and few individuals had more than 2.5 mm of ARR. In the patients with aligners, the most susceptible teeth were maxillary incisor and mandibular central incisor, followed by mandibular lateral incisor, maxillary canine, and mandibular canine. This is consistent with the previous studies [19, 21]. It has been reported that there was 2 mm ARR on the upper incisors in the periapical, panoramic, and cephalometric radiographs after 14 months of clear aligners treatment [22]. The current study indicated there was only 0.13 mm ARR in patients with clear aligners after about 22 months treatment. The differences of findings among those studies were mainly because of the different methodologies, such as sample size, clinical characteristics, imaging tools and measurement methods.

Most of those previous studies on orthodontic ARR was performed using two-dimensional radiography, such as panoramic and periapical radiographs. CBCT has shown advantages in the accuracy and efficacy for diagnosis and measurement of root resorptio n[23]. A number of previous studies used CBCT to evaluate the ARR in patients with fixed appliances and found the frequency of ARR was more than 70% in incisor s[9]. Another recent publication comparing the ARR of upper incisors between Smart Track aligners and fixed orthodontic appliances using CBCT reported that the cases treated with fixed appliances showed significantly higher root resorption than those treated with clear aligners [24]. This is similar with the current study findings. In addition to the advantages of CBCT for assessing ARR, it is also important to note that the radiation of CBCT can be 1.5 to 33 times higher than that of the traditional panoramic radiography [25], therefore future studies can consider to use the localized and limited field of view CBCT that provides the information needed to minimize patient’s exposure to radiation and expense for radiography [26].

Root resorption during orthodontic treatment is usually recognized as an orthodontically induced inflammatory root resorption [2], which is based on a sterile inflammatory process and initiated by orthodontic force application [8, 27]. Many factors have been found to influence the orthodontic root resorption, including genetics [28], ethnicity [19], systemic diseases and allergic constitution [29], gender and age [7, 30], treatment time [31], as well as the type (continuous or intermittent) and magnitude of orthodontic forces [32]. It has been found that heavy force used is associated with the prevalence of ARR [20], and an increase of force used is associated with the severity of ARR [33, 34]. Intermittent force resulted in less ARR than continuous force [32, 35] because the intermittent force provided cementum with the time to heal [36]. Compared with fixed appliances, the clear aligners have been considered to potentially deliver relatively lighter forces, intermittent treatment process, and stable force control using computer-aided technology, which may contribute to the less ARR in patients with clear aligners than that in patients with fixed appliances [12].

Negative values of ARR (an increase in the root length after treatment) were reported in the present study as well as the previous studies [5, 9, 37]. Though random error in the measurement should be taken into account, the biological variability, especially in the growing young individuals, may also contribute to that [5]. To minimize the measuring error, the choice and mark of reference points and the parameters of measuring software, which could potentially affect the reproducibility of measurement, should be studied and normalized in the future.

## Conclusion

Prevalence and severity of ARR on CBCT in patients treated with clear aligners was less than those in patients treated with fixed appliances.

## Data Availability

The raw data is present in the CBCT software of our university clinic.

## Abbreviations

ABO: American Board of Orthodontics
ARR: Apical root resorption
CBCT: Cone beam computed tomography
DI: Discrepancy index
